# Supplementary material for: The Effect of Diffuse Liver Diseases on the Occurrence of Liver Metastases in Cancer Patients: A Systematic Review and Meta-Analysis
Source: Cancers (Basel). 2021 May 7;13(9):2246. doi: 10.3390/cancers13092246 (PMC8124499; doi:10.3390/cancers13092246)
Supplement: Supplementary file 1 [file cancers-13-02246-s001.zip › cancers-1195010-supplementary.pdf]

cancers

MDPI

Systematic Review

The Effect of Diffuse Liver Diseases on the Occurrence of Liver Metastases in Cancer Patients: A Systematic Review and Meta-Analysis

Filippo Monelli [1,2], Giulia Besutti [1,2,*], Olivera Djuric [3,4], Laura Bonvicini [3], Roberto Farì [5], Stefano Bonfatti [5], Guido Ligabue [5], Maria Chiara Bassi [6], Angela Damato [7], Candida Bonelli [7], Carmine Pinto [7], Pierpaolo Pattacini [2] and Paolo Giorgi Rossi [3]

[1] Clinical and Experimental Medicine PhD Program, University of Modena and Reggio Emilia, 41124, Modena, Italy; filippo.monelli@ausl.re.it
[2] Radiology Unit, Department of Diagnostic Imaging and Laboratory Medicine, AUSL-IRCCS di Reggio Emilia, 42123, Reggio Emilia, Italy; pierpaolo.pattacini@ausl.re.it
[3] Epidemiology Unit, AUSL- IRCCS di Reggio Emilia, 42123 Reggio Emilia, Italy; olivera.djuric@ausl.re.it (O.D.); laura.bonvicini@ausl.re.it (L.B.); paolo.giorgirossi@ausl.re.it (P.G.R.)
[4] Center for Environmental, Nutritional and Genetic Epidemiology (CREAGEN), Section of Public Health, Department of Biomedical, Metabolic and Neural Sciences, University of Modena and Reggio Emilia, 41124, Modena, Italy
[5] Radiology Unit, AOU Policlinico di Modena, University of Modena and Reggio Emilia, 41124, Modena, Italy; roberto.fari2@gmail.com (R.F.); bonfste@gmail.com (S.B.); guido.ligabue@unimore.it (G.L.)
[6] Medical Library, AUSL- IRCCS di Reggio Emilia, 42123 Reggio Emilia, Italy; mariachiara.bassi@ausl.re.it
[7] Oncology Department, AUSL- IRCCS di Reggio Emilia, 42123 Reggio Emilia, Italy; Angela.damato@ausl.re.it (A.D.); candida.bonelli@ausl.re.it (C.B.); carmine.pinto@ausl.re.it (C.P.)
* Correspondence: giulia.besutti@ausl.re.it

Citation: Monelli, F.; Besutti, G.; Djuric, O.; Bonvicini, L.; Far, R.; Bonfatti, S.; Ligabue, G.; Bassi, M.C.; Damato, A.; Bonelli, C.; et al. The Effect of Diffuse Liver Diseases on the Occurrence of Liver Metastases in Cancer Patients: A Systematic Review and Meta- Analysis. *Cancers* **2021**, *13*, 2246. https://doi.org/10.3390/cancers13092246

Academic Editor: Christoph Reissfelder

Received: 7 April 2021
Accepted: 3 May 2021
Published: 7 May 2021

Publisher's Note: MDPI stays neutral with regard to jurisdictional claims in published maps and institutional affiliations.

Copyright: © 2021 by the authors. Licensee MDPI, Basel, Switzerland. This article is an open access article distributed under the terms and conditions of the Creative Commons Attribution (CC BY) license (http://creativecommons.org/licenses/by/4.0/).

Table S1. Reasons for exclusion.

	Author and Year	Reason for Exclusion
1	Peng 2015	No outcome of interest
2	Sabbagh 2015	No outcome of interest
3	Abdalla 2008	No outcome of interest
4	Parkin 2014	No outcome of interest
5	Parkin 2012	No outcome of interest
6	Pathak 2010	No outcome of interest
7	Jie You 2015	No outcome of interest
8	Viganò 2013	No outcome of interest
9	Kampfenkel 2011	No outcome of interest
10	Khan 2009	No outcome of interest
11	Song 2001	Was not possible to extract outcomes (synchronous and metachronous liver metastases counted together)
12	Han 2017	Was not possible to extract outcomes (no details on the timing of metachronous liver metastasis occurrence)
13	Zhao 2018	Was not possible to extract outcomes (no details on the timing of metachronous liver metastasis occurrence)
14	Chen 2016	Follow-up under 12 months for pancreatic cancer
15	Qiu 2011	Follow-up under 24 months for colorectal cancer, and was not possible to extract outcomes (synchronous and metachronous liver metastases counted together)
